# Increased susceptibility of cystic fibrosis airway epithelial cells to ferroptosis

**DOI:** 10.1186/s40659-021-00361-3

**Published:** 2021-12-13

**Authors:** Pramila Maniam, Ama-Tawiah Essilfie, Murugan Kalimutho, Dora Ling, David M. Frazer, Simon Phipps, Gregory J. Anderson, David W. Reid

**Affiliations:** 1 Immunology Department, QIMR Berghofer Medical Research Institute, Brisbane, Australia; 2 Cell and Molecular Biology Department, QIMR Berghofer Medical Research Institute, Brisbane, Australia; 3 School of Chemistry and Molecular Bioscience, University of Queensland, St Lucia, Australia; 4 Adult Cystic Fibrosis Centre, The Prince Charles Hospital, Chermside, Australia; 5 Lung Inflammation and Infection Laboratory, Immunology Department, QIMR Berghofer Medical Research Institute, Herston, QLD 4003 Australia

**Keywords:** Ferroptosis, Lipid peroxidation, Erastin, Iron, Cystic fibrosis, Airway epithelial cells

## Abstract

**Background:**

Defective chloride transport in airway epithelial cells (AECs) and the associated lung disease are the main causes of morbidity and early mortality in cystic fibrosis (CF). Abnormal airway iron homeostasis and the presence of lipid peroxidation products, indicative of oxidative stress, are features of CF lung disease.

**Results:**

Here, we report that CF AECs (IB3-1) are susceptible to ferroptosis, a type of cell death associated with iron accumulation and lipid peroxidation. Compared to isogenic CFTR corrected cells (C38), the IB3-1 cells showed increased susceptibility to cell death upon exposure to iron in the form of ferric ammonium citrate (FAC) and the ferroptosis inducer, erastin. This phenotype was accompanied by accumulation of intracellular ferrous iron and lipid peroxides and the extracellular release of malondialdehyde, all indicative of redox stress, and increased levels of lactate dehydrogenase in the culture supernatant, indicating enhanced cell injury. The ferric iron chelator deferoxamine (DFO) and the lipophilic antioxidant ferrostatin-1 inhibited FAC and erastin induced ferroptosis in IB3-1 cells. Glutathione peroxidase 4 (GPX4) expression was decreased in IB3-1 cells treated with FAC and erastin, but was unchanged in C38 AECs. Necroptosis appeared to be involved in the enhanced susceptibility of IB3-1 AECs to ferroptosis, as evidenced by partial cell death rescue with necroptosis inhibitors and enhanced mixed lineage kinase domain-like (MLKL) localisation to the plasma membrane.

**Conclusion:**

These studies suggest that the increased susceptibility of CF AECs to ferroptosis is linked to abnormal intracellular ferrous iron accumulation and reduced antioxidant defences. In addition, the process of ferroptotic cell death in CF AECs does not appear to be a single entity and for the first time we describe necroptosis as a potential contributory factor. Iron chelation and antioxidant treatments may be promising therapeutic interventions in cystic fibrosis.

**Graphical Abstract:**

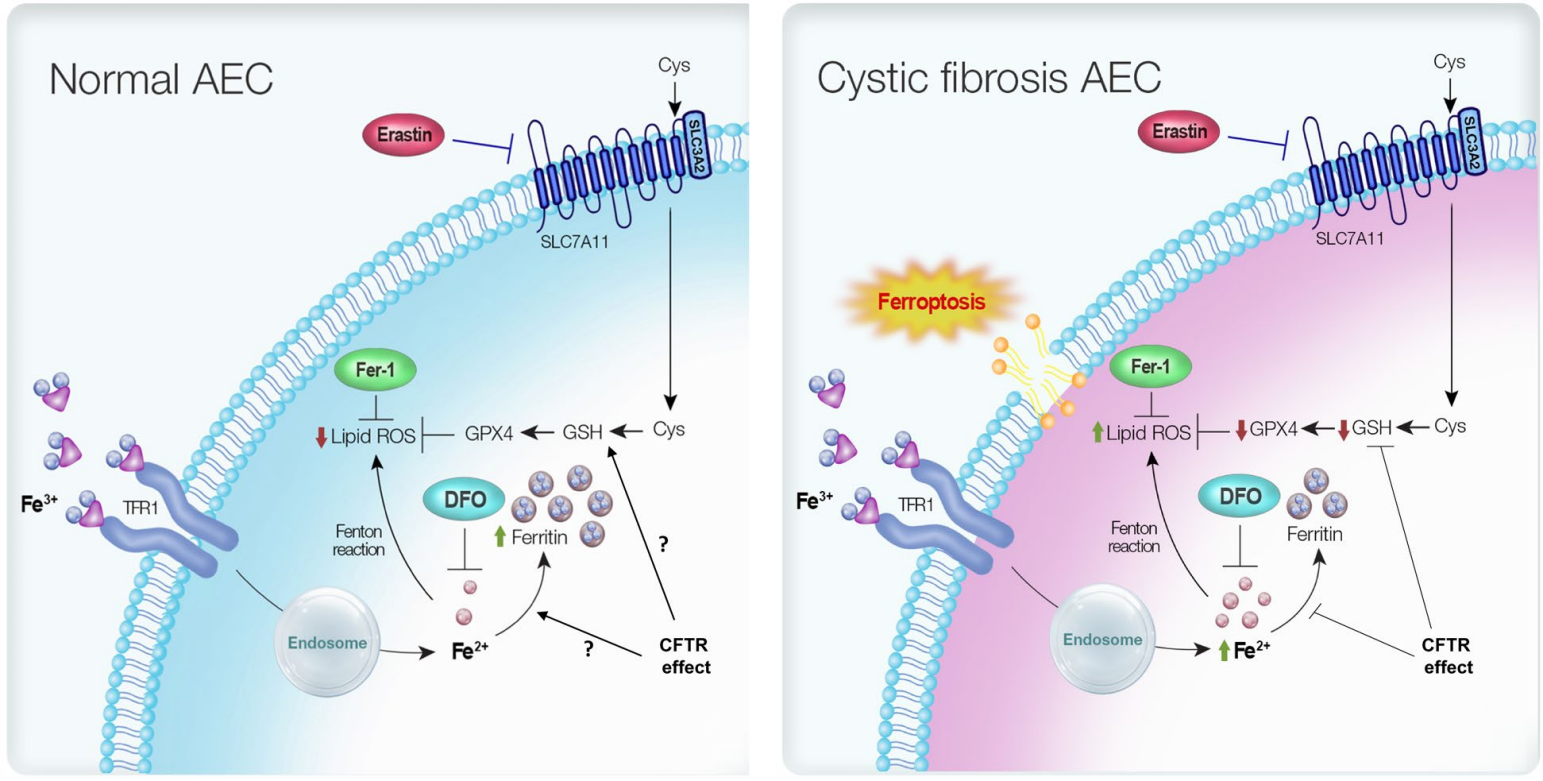

**Supplementary Information:**

The online version contains supplementary material available at 10.1186/s40659-021-00361-3.

## Background

Cystic fibrosis (CF) is an autosomal recessive condition caused by mutations in the cystic fibrosis transmembrane conductance regulator (*CFTR*) gene, which encodes a chloride ion channel [1]. Mutations in *CFTR* lead to abnormal chloride ion transport in mucus and sweat producing cells, mainly affecting epithelial cells of the respiratory and gastrointestinal tracts [2]. Lung disease with progressive bronchiectasis and airway suppuration remains the main cause of morbidity and premature mortality in patients with CF [3]. The respiratory system undergoes a vicious cycle of infection, inflammation and remodelling of lung tissue, with oxidative stress as a major contributing factor to destruction of the CF lungs [4]. Lipid peroxidation products are elevated in CF lungs, suggestive of oxidative stress [5–8]. Reduced levels of glutathione (GSH), which is a major antioxidant defence in the epithelial lining fluid of the lung have been described, which is probably multifactorial, but primarily related to dysfunctional CFTR that is involved in the extracellular transport of GSH [9–11].

Ferroptosis, an iron-dependent mode of cell death caused by the accumulation of lipid peroxides, is increasingly recognised as an important process in several pathologies including cancer, infection and chronic inflammation [12, 13]. Excess iron, or iron that is not safely compartmentalised within cells can promote the production of reactive oxygen species (ROS) through the Fenton reaction, which in turn promotes intracellular lipid peroxidation and ultimately ferroptosis. Cellular antioxidant defences are critically important for preventing ferroptosis, as evidenced by the observation that ferroptosis is initiated by inhibition of the cystine/glutamate antiporter, system ${\text{x}}_{{\text{c}}}^{ - }$ which mediates the transport of extracellular cystine into cells where it is used for GSH biosynthesis [12, 14]. The glutathione peroxidase (GPX) family of enzymes, particularly GPX4, are key modulators of lipid peroxides in cells and use GSH as a co-substrate to reduce lipid peroxides to the corresponding alcohol [15]. Inactivation of GPX4 results in the accumulation of lipid peroxides and cell death by ferroptosis [16]. The system ${\text{x}}_{{\text{c}}}^{ - }$/GSH/GPX4 axis appears pivotal in the prevention of ferroptotic cell death under normal homeostatic conditions. In vitro studies have demonstrated that iron chelators or lipophilic antioxidants such as vitamin E/α-tocopherol and ferrostatin-1 (Fer-1) can also prevent ferroptosis, by targeting either the precipitant (redox active iron), or enhancing cellular defence mechanisms [15].

Although there are data emerging on the role of ferroptosis in respiratory diseases such as asthma, chronic obstructive pulmonary disease (COPD) and tuberculosis, participation of ferroptosis in CF is still poorly characterised. Ferroptosis may be particularly relevant to CF given the documented deficiency in GSH defences and evidence of abnormal iron homeostasis in the CF lung, which together create the milieu for the “perfect storm” of redox stress and promotion of ferroptosis [5, 17–19].

Our study investigated the involvement and mechanism of ferroptosis in CF airway epithelial cells (AECs). Here, we show that CF AECs are more susceptible to cell death by ferroptosis than their cognate wild-type cells, and this is associated with increased intracellular labile iron content, decreased GSH and GPX4 levels, and accumulation of lipid peroxides. Combined treatment with an iron chelator and ferrostatin-1 attenuated ferroptosis in the CF AECs. The enhanced susceptibility of CF AECs to ferroptosis is consistent with the published literature suggesting that iron homeostasis is disrupted in the CF lung [20, 21]. This study provides rationale for the development of iron- and lipid peroxidation-targeting therapies in cystic fibrosis.

## Materials and methods

### Cell culture, antibodies and reagents

C38 and IB3-1 human bronchial epithelial (HBE) cell lines were obtained from Queensland Children’s Medical Research Institute, Australia. IB3-1 cells were derived from a compound heterozygote CF patient with one ΔF508 allele and one W1282X nonsense mutation allele. This phenotype has been corrected in the C38 cell line by the introduction of wild-type (WT) CFTR using an adenosine-associated viral vector. C38 and IB3-1 cells were seeded onto collagen (30 µg/mL) (Sigma-Aldrich, C3867) and bovine serum albumin (BSA) (10 µg/mL) (Sigma-Aldrich, A8412) coated flasks and maintained in Dulbecco’s Modified Eagle Medium (DMEM) (Gibco, 11320033) containing 10% fetal bovine serum (FBS) (Sigma-Aldrich, A8412). Cells were routinely screened for the presence of mycoplasma by QIMR Berghofer Scientific Services. Human colorectal adenocarcinoma cells (Caco-2) were maintained in DMEM cell culture medium supplemented with 5% FBS and 1% nonessential amino acids (NEAA) (Gibco, 11140050).

The antibodies used in this study were rabbit anti-GPX4, rabbit anti-nuclear receptor coactivator 4 (NCOA4), rabbit anti-ferritin, rabbit anti-transferrin receptor 1 (TFR1), mouse anti-β-actin, rabbit anti-mixed lineage kinase domain-like pseudokinase (MLKL), HRP-conjugated goat anti-mouse IgG secondary antibody, HRP-conjugated goat anti-rabbit IgG secondary antibody and Alexafluor goat anti-rabbit 546. Antibodies were used at the dilutions specified in the manufacturer’s instructions, and details are provided in Additional file 1: Table S1.

Other reagents used included ferric ammonium citrate (FAC) (Sigma-Aldrich, F5879) erastin (Sigma-Aldrich, E7781), deferoxamine (DFO) (DBL Pharma), ferrostatin-1 (Fer-1) (Sigma-Aldrich, 0583), necrosulfonamide (NSA) (Calbiochem, 480073), necrostatin-1 (Nec-1s) (Selleckchem, 8641), z-VAD-FMK (zVAD) (Becton Dickinson, 550377), *N*-acety-l-cysteine (NAC) (Sigma-Aldrich, A9165), wortmannin (Sigma-Aldrich, W1628), β-mercaptoethanol (Sigma-Aldrich, 3148), Hoescht 33342 (Invitrogen, 1399) and DAPI (Thermo Scientific, 62247).

### Cell death kinetics assessment by IncuCyte

Cell death was assessed by incubating cells with SYTOX™ Green nucleic acid stain (125 nM, Thermo Fisher Scientific, S7020). Cells were imaged at 2 h intervals for 48 h using the IncuCyte^®^ ZOOM live cell analysis system (Essen BioScience). Cell death was measured by counting maximum SYTOX™ Green positive cells using the IncuCyte^®^ ZOOM live cell analysis software. SYTOX™ Green positive counts in each well were normalised using starting cell confluence inferred from phase-contrast images acquired in parallel as a metric.

### Measurement of cell viability and cytotoxicity

Cell viability was assessed by a colorimetric method using CellTiter 96^®^ AQueous One Solution Cell Proliferation Assay (3-(4,5-dimethylthiazol-2-yl)-5-(3-carboxymethoxyphenyl)-2-(4-sulfophenyl)-2H-tetrazolium, MTS) (Promega, G3582) according to the manufacturer’s instructions. Cytotoxicity was assessed using a commercially available colorimetric method according to the manufacturer’s protocol (Pierce, 88953). This method is based on the relative release of lactate dehydrogenase (LDH) into the cell culture medium, and cytotoxicity is expressed as the percentage increase in LDH release in the test group relative to the vehicle-treated control group. The following formula was used to calculate percentage cytotoxicity [22]:

$$\begin{gathered} \%{\text{Cytotoxicity}} = {\frac{{{\text{LDH activity of samples }} - {\text{spontaneous LDH release control*}}}}{{{\text{Maximum LDH release control}}^{{{\# }}} - {\text{spontaneous LDH release control*}}}} } \times 100, \hfill \\ *{\text{water}}\;{\text{treated}} \hfill \\^{{{\# }}} {\text{lysis buffer treated}}{.} \hfill \\ \end{gathered}$$

### Measurements of malondialdehyde and iron levels

Intracellular malondialdehyde (MDA) content was measured using a fluorometric lipid peroxidation assay kit (Abcam, 118970) following the manufacturer’s instructions. Total iron (ferric and ferrous) and ferrous (Fe^2+^) iron in cells were analysed using an iron assay kit (Abcam, 83366) according to the manufacturer’s protocols.

### Lipid peroxidation assessment

Lipid peroxidation was measured using the C11-BODIPY 581/591 lipid peroxidation sensor (Invitrogen, D3861) as described previously [23]. Briefly, C38 and IB3-1 cells were seeded at a density of 5 000 cells per well in a 96-well microplate. Cells were incubated for 30 min with Hoechst 33342 and C11-BODIPY 581/591 (1 µM) in growth medium. The medium was then removed and the cells were washed with phosphate buffered saline (PBS) three times. Imaging was carried out using EVOS FL Auto 2 inverted fluorescence microscope using a 20X objective and filters for Texas red (590 nm), FITC (510 nm) and DAPI channels. The signal was then quantitated by QuPath image analysis software [24] and the ratios of the signal from the 590/510 channels were used to quantify lipid peroxidation in cells.

### Real-time PCR (qPCR) analysis

Total RNA was isolated using Purelink RNA mini kit (Invitrogen, 12183018A), then reverse-transcribed into complementary DNA using M-MLV reverse transcriptase (Invitrogen, 28025013). qPCR analysis was carried out using the QuantStudio^®^ 5 System (Applied Biosystems) real time PCR instrument and software. A list of primer sequences is provided in Additional file 1: Table S2.

### Glutathione assay

Intracellular glutathione levels were measured using the glutathione colorimetric detection kit (Invitrogen, EIAGSHC). Briefly, cell lysates were prepared by washing cell pellets in ice-cold PBS and resuspending them in ice-cold 5% 5-Sulfosalicylic Acid (SSA) (Sigma-Aldrich, S2130) solution at 1 × 10^6^ cells/mL. Cells were lysed by multiple freeze–thaw cycling, then incubated for 10 min at 4 °C and centrifuged at 14,000 rpm for 10 min to collect supernatant for further analysis according to the manufacturer’s protocol.

### Western blotting

Cells grown in 6-well plates were lysed in RIPA buffer supplemented with complete protease (Roche, 4693116001) and phosphatase (Roche, 4906845001) inhibitor cocktails. Total protein was quantitated by bicinchoninic acid (BCA) protein assay [25]. Western blotting was performed as previously described [26]. Briefly, equivalent amounts (30 µg) of protein samples were resolved on 10–15% SDS-PAGE gels [27] and electro-transferred onto PVDF membranes (Merck, IPFL00010). Then the membrane was blocked with 5% skim milk and incubated with primary antibodies overnight at 4 °C with gentle agitation. Blots were then incubated with secondary antibody for one hour at room temperature. Detection of immune-reactive bands was carried out using ECL western blot detection reagents (PerkinElmer, NEL103001EA). Western blots were documented using the ChemiDoc™ gel imaging system (Bio-Rad). Densitometry analysis was carried out using Image Lab image acquisition and analysis software (Bio-Rad).

### Immunofluorescence

Cells on 8-well chamber slides (Thermo Scientific, NUN154534) were washed with PBS pH 7.4 and fixed in 4% paraformaldehyde (PFA) in PBS for 15 min at room temperature. Cells were washed with PBS three times for 5 min, then blocked with 2% bovine serum albumin (BSA) in PBS for one hour. Subsequently, cells were incubated with primary antibody (1:200 dilution of MLKL) at 37 ºC in a humidified chamber for one hour. Cells were washed with PBS and then incubated with secondary antibody supplemented with DAPI at 37 °C in a humidified chamber for 30 min. Cells were washed with PBS and then mounted with a coverslip by adding ProLong™ Gold Antifade Mountant (Invitrogen, P36934). The slide was cured at room temperature in the dark for 24 h and then stored at 4 °C until being imaged. Slides were imaged using a DeltaVision^®^ Deconvolution microscope (Applied Precision). Typically, for each independent experiment, 5–7 randomly selected fields were captured per treatment group. To ensure consistent signal intensities across independent experiments, the same excitation, emission and camera settings were used throughout this study.

### Statistical analysis

Statistical analyses were performed using Student’s *t*-test or one-way analysis of variance (ANOVA) followed by Tukey’s multiple comparison test. Quantitative data are presented as the mean ± standard error of the mean (SEM). Differences were considered significant at *p < 0.05, **p < 0.01 and ***p < 0.001.

## Results

### IB3-1 cystic fibrosis cells are more susceptible to cell death by ferroptosis

**Fig. 1:**
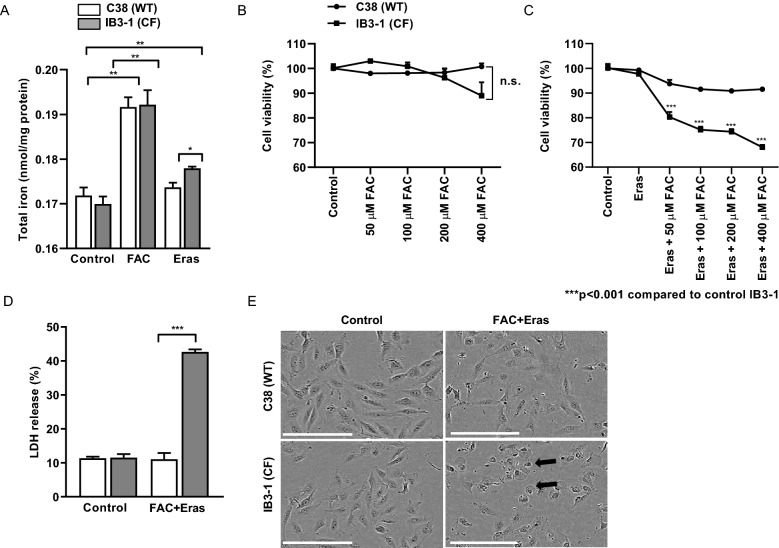
IB3-1 cystic fibrosis AECs are susceptible to cell death caused by FAC and erastin. AECs were treated with FAC (100 µM) or erastin (Eras) (10 µM) and total iron was assayed (**A**). AECs were treated with FAC (50–400 µM) (**B**) and/or erastin (10 µM) (**C**) for 12 h and cell viability was assessed by MTS assay. Cells were treated with 100 µM FAC and 10 µM erastin for 12 h and LDH release was measured (**D**) or cells were imaged using IncuCyte Zoom instrument (**E**). Scale bar represents 150 µm. Data are expressed as mean ± SEM. *p < 0.05, **p < 0.01 and ***p < 0.001 for statistical analysis of the indicated groups

In order to determine the susceptibility of the C38 and IB3-1 cell lines to iron loading, we first measured the levels of intracellular total non-heme iron in the cells. We found that both cell lines had the same level of total iron at baseline (Fig. 1A). Iron treatment in the form of ferric ammonium citrate (FAC) (100 µM) increased the levels of total iron in both cell lines to the same extent at 8 h post-incubation. Erastin is a small molecule which is known to inhibit system ${\text{x}}_{{\text{c}}}^{ - }$, thus reducing the uptake of the amino acid cysteine required for synthesis of the antioxidant glutathione [12]. In the presence of erastin (10 µM), IB3-1 cells showed significantly more iron levels compared to the C38 cells at 8 h post-incubation (Fig. 1A).

We also assessed the effect of varying concentrations of FAC on cell viability. There were no apparent changes in cell viability in either cell line treated with up to 400 µM FAC (Fig. 1B). We then tested if these cells were more prone to ferroptosis. In both IB3-1 and C38 cells, treatment with erastin alone did not decrease cell viability (Fig. 1C). However, in IB3-1 but not C38 cells, treatment with both erastin and FAC led to a dose-dependent (from 50 to 400 µM FAC) decrease in cell viability (Fig. 1C).

To confirm this finding, we assessed cytotoxicity by measuring LDH in the cell culture medium. In contrast to control C38 cells, FAC (100 µM) and erastin (10 µM) induced a four-fold increase in LDH release in IB3-1 cells at 12 h post-treatment (Fig. 1D). In addition, morphologically, FAC and erastin treated cells showed numerous dead cells compared to C38 cells (Fig. 1E). These findings indicate that although FAC or erastin alone did not affect cell viability, combined FAC and erastin treatment is cytotoxic to IB3-1 cells. Taken together, these data demonstrate that IB3-1 CF cells have increased susceptibility to cell death by ferroptosis.

### Ferroptotic cell death is prevented in IB3-1 CF AECs by DFO or Fer-1

**Fig. 2:**
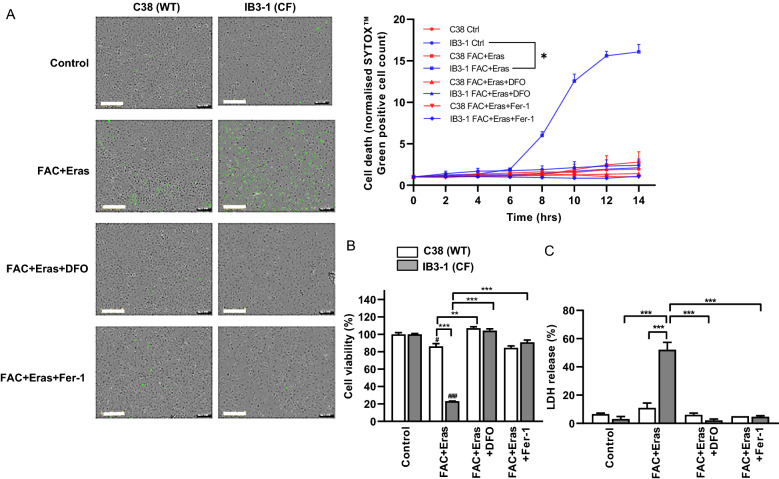
DFO and Fer-1 prevented ferroptotic cell death in IB3-1 cells. AECs were treated with FAC (100 µM) and erastin (Eras) (10 µM) in the presence or absence of DFO (100 µM) or Fer-1 (2 µM) for 12 h. Cell death kinetics were assessed by IncuCyte^®^ ZOOM system using SYTOX™ Green cell death marker. Representative images taken at 12 h post-treatment and cell death kinetics plot generated by normalising SYTOX positive cell count to starting cell confluence are shown (**A**). Cell viability was assessed by MTS assay (**B**) and cytotoxicity levels were assessed by measuring LDH release (**C**). Scale bar represents 300 µm. Data are expressed as mean ± SEM. *p < 0.05, **p < 0.01 and ***p < 0.001 for statistical analysis of the indicated groups

In order to confirm that FAC and erastin co-exposure induces ferroptosis in the CF cells, we co-incubated the treated cells with the iron chelator DFO or lipophilic antioxidant Fer-1. DFO chelates ferric iron to limit iron-mediated ROS production by the Fenton reaction, which promotes lipid peroxidation in ferroptosis [28]. Fer-1 is a lipophilic antioxidant that inhibits the formation of lipid peroxides, which are cytotoxic radicals [29]. Monitoring of cell death in real-time demonstrated accumulation of the SYTOX™ green cell death marker in IB3-1 cells (but not C38 cells) commencing at 8 h post-treatment with FAC and erastin, with the maximum signal reached at 12 h (Fig. 2A). The FAC and erastin-induced increase in SYTOX™ green signal was ablated following treatment with either DFO or Fer-1. Consistent with these findings, DFO and Fer-1 prevented cell death caused by FAC and erastin in IB3-1 cells, as shown by the assessment of cell viability (Fig. 2B). Moreover, high levels of LDH release as a marker of cell injury in IB3-1 cells in response to FAC and erastin exposure were significantly decreased upon treatment with either DFO or Fer-1 (Fig. 2C).

In order to confirm that promotion of cell death by FAC and erastin is related to the loss of CFTR function, we investigated the effects of the CFTR inhibitors CFTR(inh)-172 and GlyH-101 on immortalised human colorectal adenocarcinoma cells (Caco-2). This cell line highly expresses CFTR and has been widely used to study the functional response of CFTR to various extracellular factors [30–32]. FAC and erastin treated Caco-2 cells did not undergo significant cell death compared to non-treated controls, unless they were also treated with a CFTR inhibitor (Additional file 1: Fig. S1A). We therefore hypothesised that CFTR inhibition in C38 cells would promote susceptibility to ferroptosis. Consistent with this notion, treatment with CFTR inhibitor predisposed to FAC and erastin induced cell death (Additional file 1: Fig S1B and C). These data suggest that CFTR functions as a potential negative regulator of ferroptosis.

### Increased lipid peroxidation as a hallmark of ferroptosis in CF cells

**Fig. 3:**
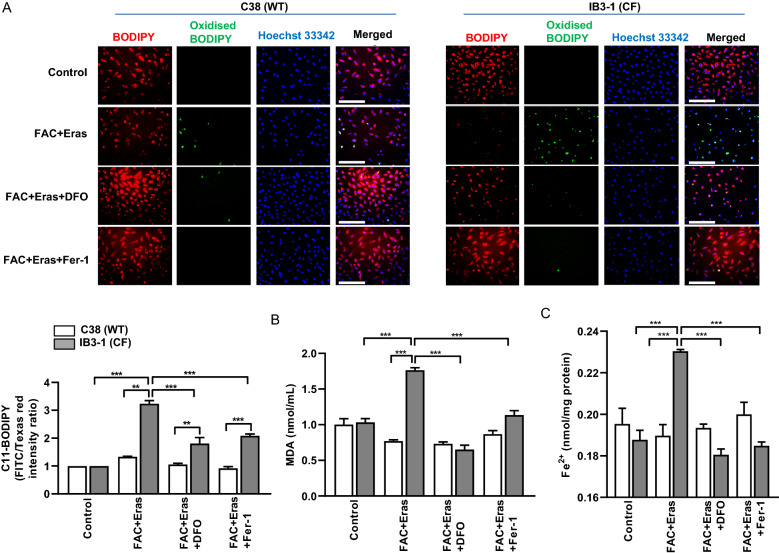
Increased lipid peroxidation and iron levels in FAC and erastin treated IB3-1 cells. AECs were treated with FAC (100 µM) and erastin (Eras) (10 µM) in the presence or absence of DFO (100 µM) or Fer-1 (2 µM) for 8 h. Lipid peroxidation were assayed in cells using the lipophilic redox-sensitive dye C11-BODIPY 581/591, which shifts its fluorescence from red to green in response to oxidation. Representative images and quantification are shown (**A**). MDA (**B**) and ferrous iron levels (**C**) were assayed. Scale bar represents 400 µm. Data are expressed as mean ± SEM. **p < 0.01 and ***p < 0.001 for statistical analysis of the indicated groups

Mechanistically, an increase in lipid hydroperoxides is the key downstream feature of ferroptosis [14]. In order to assess the extent of lipid peroxidation, we used BODIPY 581/591 C_11_ fatty acid, which is a lipophilic lipid peroxidation sensor that shifts from red to green upon oxidation of the polyunsaturated phenylbutadiene segment of the fluorophore [33]. This oxidation-dependent emission shift enables fluorescence ratio imaging of lipid peroxidation in live cells. Quantification of the signals obtained following FAC and erastin exposure revealed that IB3-1 cells had a threefold increase in oxidised C11-BODIPY signal intensity compared to non-treated controls (Fig. 3A), and this response was significantly decreased in the presence of DFO or Fer-1. In contrast, C38 cells did not show any significant increase in oxidised C11-BODIPY signals with FAC and erastin treatments as compared to its control. These findings suggest that IB3-1 CF cells have an increased tendency for lipid peroxidation compared to C38 cells.

Malondialdehyde (MDA) is a highly reactive and toxic by-product of lipid peroxide degradation and is therefore used as a surrogate marker for lipid peroxidation. There was no basal level difference in MDA levels in IB3-1 and C38 (Fig. 3B). However, IB3-1 cells exhibited twofold higher MDA production than C38 cells upon FAC and erastin treatment and this increase was attenuated by either DFO or Fer-1 treatment.

An important driver of lipid peroxide formation and thus MDA production is the presence of reactive ferrous iron (Fe^2+^), which catalyses the formation of free radicals that damage cell lipids via Fenton chemistry [16]. Both ferric and ferrous iron have been shown to be abundant in the CF lung and are correlated with disease severity [20]. To investigate the role of ferrous iron, we assessed the relative content of ferrous iron in FAC and erastin treated IB3-1 CF and cognate C38 cells and found that the IB3-1 cells exhibited significantly increased levels of ferrous iron accumulation following FAC and erastin treatment, which was not apparent in C38 cells (Fig. 3C). Collectively, these data confirm the involvement of lipid peroxidation with increased production of the by-product MDA, and high levels of ferrous iron which are consistent with the increased susceptibility to ferroptotic cell death in CF cells.

### Altered levels of glutathione and markers of ferroptosis in FAC and erastin treated IB3-1 cells

**Fig. 4:**
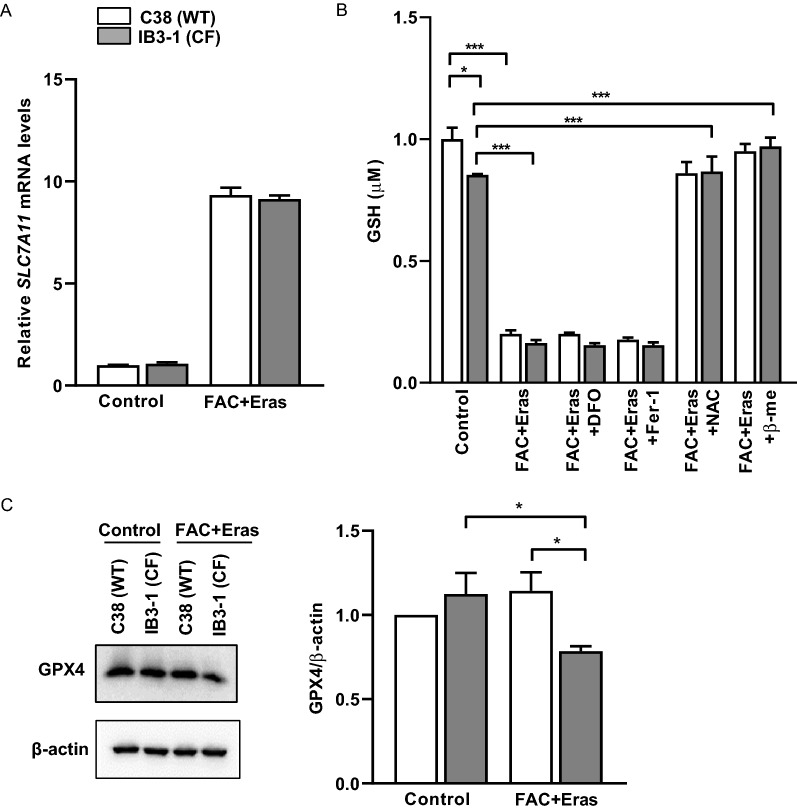
Altered expression levels of ferroptosis markers and GSH levels in FAC and erastin treated cells. AECs were treated with FAC (100 µM) and erastin (Eras) (10 µM) for 8 h. *SLC7A11* gene expression (**A**), glutathione levels (**B**) and representative (n = 3) immunoblot image for GPX4 and quantification (**C**) were assessed. Data are expressed as mean ± SEM. *p < 0.05 and ***p < 0.001 for statistical analysis of the indicated groups

Erastin induces ferroptosis by irreversibly inhibiting SLC7A11, which is a key component of system ${\text{x}}_{{\text{c}}}^{ - }$ [34]. Dixon et al. have suggested that erastin binding to SLC7A5 interferes with cysteine uptake by system ${\text{x}}_{{\text{c}}}^{ - }$ [12]. In turn, this may lead to a compensatory transcriptional upregulation of *SLC7A11*. Consistently, Dixon et al. observed substantial upregulation of *SLC7A11* in erastin-treated HT-1080 cells. Similarly, we observed upregulation of *SLC7A11* mRNA in both C38 and IB3-1 cells following FAC and erastin treatment (Fig. 4A).

Erastin binding to the SLC7A5/SLC3A2 component of system ${\text{x}}_{{\text{c}}}^{ - }$ interferes with cystine uptake [12, 35], which is essential for glutathione (GSH) synthesis. We therefore next assessed levels of GSH in the IB3-1 and C38 cell lines. Basal GSH levels were significantly lower in IB3-1 cells compared to C38 cells and were further decreased upon FAC and erastin exposure (Fig. 4B). Treatment with either DFO or Fer-1 failed to prevent the decrease in GSH levels, which was expected since the effects of iron chelation (DFO) and inhibition of lipid peroxide formation (Fer-1) are elicited downstream of GSH production. Interestingly, addition of the glutathione donor *N*-acetyl cysteine (NAC) or the cystine donor β-mercaptoethanol was able to restore the levels of GSH to those of control cells. This confirms that disruption of system ${\text{x}}_{{\text{c}}}^{ - }$ and cystine uptake by erastin affects intracellular GSH production in both cell lines.

GPX4 catalyses the reduction of lipid hydroperoxides by GSH and is considered an important modulator of ferroptosis. Intriguingly, although GSH levels were reduced by FAC and erastin treatment in both IB3-1 and cognate C38 cells, western blot analysis revealed that GPX4 expression remained unchanged in the C38 cells whereas it decreased significantly in IB3-1 cells (Fig. 4C; Additional file 1: Fig. S2). These observations suggest that part of the survival propensity of C38 cells may be due to an enhanced capacity to continue to prevent lipid hydroperoxides formation (Fig. 3A), even in the setting of reduced levels of GSH.

An important control mechanism of intracellular iron homeostasis is ferritinophagy, which involves the degradation of the iron-storage protein ferritin by nuclear receptor coactivator 4 (NCOA4). In order to assess if ferritinophagy contributes to the process of ferroptosis, we assessed the intracellular levels of NCOA4 and ferritin in IB3-1 and C38 cell lines. Interestingly, intracellular ferritin was increased in the IB3-1 cells at baseline compared to the C38 cells (Additional file 1: Fig. S3). Intracellular ferritin levels significantly increased in the C38 cells following FAC and erastin treatment compared to non-treated controls (Additional file 1: Fig. S3). In contrast, there was no significant change in intracellular ferritin concentrations observed in the FAC and erastin-treated IB3-1 cells. NCOA4 expression was found to be reduced in both cell lines upon FAC and erastin treatment. Based on these observations, we conclude that ferritinophagy is unlikely to be involved in the process of ferroptosis in the CF cells. We also assessed TFR1 expression as an important iron import mechanism to determine whether it may potentially be involved in disrupted cell iron homeostasis as part of ferroptosis, but we found no difference in the expression of TFR1 upon FAC and erastin treatment in either C38 or IB3-1 cells (Additional file 1: Fig. S3).

### Iron and erastin treatments induce necroptosis in CF cells

**Fig. 5:**
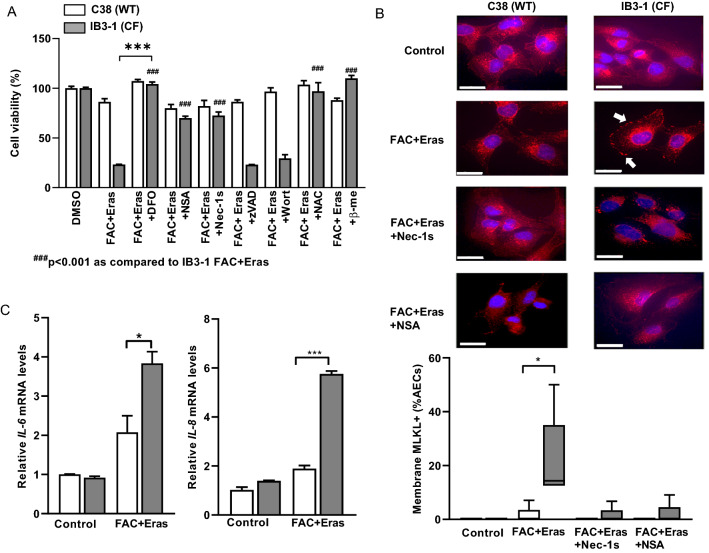
Necroptosis is partly involved in IB3-1 cell death induced by FAC and erastin. Cells were treated with FAC (100 µM) and erastin (Eras) (10 µM) in the presence or absence of DFO (100 µM), NSA (10 µM), Nec-1s (50 µM), zVAD (50 µM), wortmannin (5 µM), NAC (10 mM) or β-mercaptoethanol (50 µM) for 12 h and cell viability was measured (**A**). MLKL membrane localisation was assessed by immunofluorescence. Representative images and quantification are shown (**B**). *IL-6* and *IL-8* mRNA levels were assessed (**C**). Scale bar represents 25 µm. Data are expressed as mean ± SEM. *p < 0.05, **p < 0.01 and ***p < 0.001 for statistical analysis of the indicated groups

We further investigated if FAC and erastin treatment induced other types of cell death, i.e. necroptosis, apoptosis and/or autophagy. Neither the addition of pan-caspase inhibitor zVAD nor the autophagy inhibitor wortmannin affected FAC and erastin-induced cell death, suggesting that apoptosis and autophagy were unlikely to be involved (Fig. 5A).

In contrast, necroptosis inhibitors necrosulfonamide (NSA) and necrostatin-1s (Nec-1s) which inhibits MLKL and RIPK1 respectively, partially (to 68–72% of control cell viability) prevented FAC and erastin-induced cell death in the IB3-1 CF cells (Fig. 5A). In addition, the glutathione donor NAC and cystine donor β-mercaptoethanol rescued the cells.

During necroptosis, receptor-interacting serine/threonine-protein kinase 1 (RIPK1) and RIPK3 interact with each other to form a functional heterodimer complex [36]. This complex phosphorylates MLKL and in turn promotes its oligomerisation. Oligomeric MLKL translocates to the plasma membrane from the cytosol, resulting in the formation of pores, and causing an inflammatory response [37]. In order to validate the involvement of necroptosis in CF cells, we stained the cells with an anti-MLKL antibody. IB3-1 cells treated with both FAC and erastin showed accumulation of MLKL signal on the plasma membrane. The appearance of MLKL on the plasma membrane was inhibited by Nec-1s and NSA, signifying the co-involvement of necroptosis in cells undergoing ferroptosis (Fig. 5B; Additional file 1: Fig. S4). To confirm the induction of inflammatory response, we assessed the expression levels of the pro-inflammatory cytokines IL-6 and IL-8 in cell lysates. Both C38 and IB3-1 CF cells demonstrated an increase in IL-6 and IL-8 with FAC and erastin, however the levels were much higher in the IB3-1 cells (Fig. 5C).

To further investigate commonalities between ferroptosis and necroptosis, we studied whether the presence of necroptosis inhibitors would prevent lipid peroxidation in the FAC and erastin treated cells. Nec-1s and NSA reduced C11-BODIPY signals by approximately 50% in FAC and erastin-treated IB3-1 cells (Additional file 1: Fig. S5). Taken together, our results suggest that FAC and erastin-induced ferroptosis in CF cells shares some common pathways with necroptosis. However, further studies are required to clarify the mechanism of cross-talk between these two pathways in cystic fibrosis.

## Discussion

In this study, we demonstrated for the first time that AECs in CF are susceptible to cell death by the iron-dependent ferroptosis pathway. Our novel findings highlight the relevance of decreased antioxidant defences, characterised by a reduction in glutathione generation in CF AECs, which appear to be related to exhaustion of glutathione peroxidase 4 (GPX4). The reduction in glutathione in the CF AECs was accompanied by increased intracellular levels of redox active ferrous iron. CF AECs also demonstrated increased baseline levels of intracellular ferritin, but an apparent reduced capacity to further up-regulate synthesis of this iron-storage protein. Interestingly, our data also suggest that ferroptosis in CF AECs is associated with necroptosis with evidence for translocation of MLKL to the plasma membrane accompanied by an enhanced inflammatory response in IB3-1 CF AECs when exposed to FAC and erastin. Consistent with the potential for shared pathways, necroptosis inhibitors were able to partially salvage CF AECs from FAC and erastin-induced ferroptosis.

Iron is a vital trace element that plays an important role in multiple biological processes in the human body. However, iron overload and disruption of cellular iron homeostatic mechanisms can generate reactive oxygen species (ROS) via the Fenton reaction and there are now numerous lines of evidence that implicate disordered iron regulation in a number of lung diseases, including CF and COPD [19, 38]. Ferroptosis is a relatively newly recognised form of cell death that is characterised by disruption of intracellular iron homeostasis and accumulation of lipid-derived ROS, which exceed the cell’s antioxidant capacity causing injury to the lipid bilayer of the cell membrane, loss of structural integrity and cell death [39]. To date, there have been no descriptions of ferroptosis occurring in the context of the pathogenesis of CF lung disease [40–42]. Thus, we demonstrated that CF AECs are susceptible to iron and erastin-induced ferroptosis, characterised by downregulated GPX4, intracellular ferrous iron accumulation, depleted GSH and lipid peroxidation, changes that were not observed in the cognate C38 cells. The ferric iron chelator DFO and radical trapping antioxidant Fer-1 were both individually able to prevent ferroptosis in CF cells. Interestingly, Fer-1 also reduced intracellular ferrous iron accumulation, which is consistent with the known chemistry of Fer-1 and its ability to complex ferrous iron (ferrostatin–iron complex) thus reducing the labile intracellular pool, which occurs as part of a catalytic cycle that serves to regenerate the Fer-1 molecule [43].

The three cornerstone events leading to ferroptosis are; abnormal iron homeostasis with resultant oxidative stress, lipid peroxidation and depleted antioxidant capacity, which predominantly manifests as reduced levels of GSH. Whilst ferroptosis has not been characterised in CF previously, published data demonstrate that all of the contributory factors required for ferroptosis are present in the CF lung. Altered iron homeostasis in the CF lung has been described [38, 44], although the mechanism linking CFTR dysfunction to abnormal iron handling remains unclear. The presence of oxidative stress in the lung in CF is very well described and plays a pivotal role in the pathogenesis of disease, and there are emerging data that demonstrate cellular lipid metabolism is also disrupted in CF [5, 45]. The propensity for ferroptosis in CF is probably directly linked to CFTR dysfunction, which is associated with reduced activity of γ-glutamylcysteine synthetase (GCS) and decreased ability to import cysteine into the cell, which are both required for GSH biosynthesis [11, 46, 47]. Consistent with this, we found that IB3-1 cells had lower intracellular levels of GSH than their cognate C38 cells at baseline and GSH levels were further reduced by FAC and erastin treatment. The inhibition of system ${\text{x}}_{{\text{c}}}^{ - }$ by erastin and prevention of cystine entry into the cell causes a reduction in GSH levels that has indirect knock-on effects, including a decrease in the synthesis of GPX4 for which GSH is an important co-substrate. GPX4 plays a pivotal role in regulating lipid peroxides within cells and the reduction in GSH and GPX4 that we demonstrate in the IB3-1 AECs following FAC and erastin treatment is likely responsible for the increased lipid peroxidation that we found, as evidenced by the increase in MDA and C11-BODIPY.

In addition to abnormal iron homeostasis and depleted GSH antioxidant defence systems, we speculate that an increased propensity to ferroptosis in CF AECs may be linked to intrinsic alterations in fatty acid metabolism. Recent studies show that abnormalities in polyunsaturated fatty acid (PUFA) metabolism secondary to CFTR dysfunction are mediated via the AMP-activated protein kinase (AMPK) signalling pathway [48]. PUFAs such as arachidonic acid and adrenic acid can be oxidised to generate lipid hydroperoxides, which then promote ferroptosis [49]. The metabolism of lipid hydroperoxides results in the production of reactive aldehydes such as MDA [50], which has previously been suggested as a new marker of oxidative stress in patients with CF due to high MDA levels in exhaled breath condensates, sputum and plasma compared to healthy controls [8]. Our study is the first to identify an association between high levels of intracellular MDA and ferroptosis in CF AECs. While the biological role of MDA in ferroptosis needs to be considered and further delineated, there is also the exciting prospect of non-invasive analysis of exhaled breath MDA levels as a measure of ferroptosis in vivo.

In our studies, we also considered the possibility that CFTR dysfunction may predispose CF AECs to other forms of cell death [40, 51]. Disruption of lung iron homeostasis appears to be involved in several lung diseases and we therefore looked for evidence of ferritinophagy, which describes a form of selective autophagy characterised by degradation of intracellular ferritin mediated by nuclear receptor coactivator 4 (NCOA4), which increases intracellular iron levels leading to accumulation of lipid ROS and ultimately cell death [52]. We demonstrated that ferritinophagy is not associated with ferroptotic cell death in IB3-1 cells as evidenced by consistently low NCOA4 levels. However, despite the absence of ferritinophagy we found that intracellular ferrous iron levels in IB3-1 cells (but not C38 cells) increased following erastin and FAC exposure, which requires further investigation as the pathways responsible for this increase in redox active iron in response to ferroptotic induction are currently unknown, but will become targets for future treatment interventions. We found increased intracellular ferritin levels in the IB3-1 cells at baseline which potentially suggests that the cells may be responding to the redox active iron present, but there was also a reduced capacity to increase ferritin synthesis when stimulated with further FAC and erastin in these cells, which suggests that the IB3-1 cells may have limited capacity beyond a certain point to combat additional increases in intracellular iron. Differential expression of ferritin-H and ferritin-L has been previously reported in the goblet, ciliated and basal subtypes air–liquid interphase (ALI) cultured CF airway epithelial cells [53], suggesting that future studies of ferroptosis using ALI cultured cells may be able to explain the increased ferritin in IB3-1 cells. Furthermore, ALI cultured cells would enable the assessment of acidic airway surface liquid (ASL) which contributes to oxidative stress in CF airways [54].

Interestingly, we found that necroptosis may be a constituent of ferroptosis, with reduced GPX4 expression being the potential common link. In a model of COPD, Yoshida et al*.* reported that in addition to ferroptosis, GPX4 was involved in the release of disease associated molecular patterns that were capable of triggering necroptosis. These findings suggest that the processes underlying ferroptosis may also be involved in additional forms of (regulated) necrotic cell death, and the participation of necroinflammatory amplification loops [55]. Confirming the co-existence of several inter-related necrotic cell death pathways will be important, as it is highly likely that they will contribute to tissue injury, inflammation and organ dysfunction in CF and other lung diseases.

The relevance of ferroptosis to disease pathogenesis and lung destruction in CF requires further investigation. However, we hypothesise that ferroptosis and the potential co-existence of necroptosis play central roles in initiation and maintenance of redox stress and chronic inflammation in the CF lung and possibly other organ systems. Demonstrating that ferroptosis is a driver of disease in CF offers the opportunity to intervene therapeutically and in this study we showed that the GSH prodrug N-acetylcysteine (NAC) was able to recover the levels of GSH back to normal and alleviate erastin-induced ferroptotic cell death. DFO was also able to prevent ferroptosis in the IB3-1 CF AECs, presumably through its ability to chelate ferric iron safely and circumvent the generation of redox active ferrous iron. The use of NAC and iron chelation therapy have both been considered in CF lung disease, but not in the context of ferroptosis and further studies are warranted.

## Conclusion

In summary, our novel findings indicate that IB3-1 cystic fibrosis AECs are much more susceptible to cell death by ferroptosis than their cognate C38 AECs, which appears underpinned by abnormal intracellular accumulation of ferrous iron, and decreased antioxidant defences, as manifested by low GSH and GPX4 levels, and unrestrained lipid peroxidation. While lipid peroxides and high iron levels have been detected in the lungs of cystic fibrosis patients, it remains unclear to what extent ferroptosis may be involved in the initiation and maintenance of disease, but better understanding of how ferroptosis contributes to the pathophysiology of cystic fibrosis lung disease is critical and may provide the impetus to new therapeutic interventions.

## Supplementary Information

**Additional file 1: Table S1**. List of antibodies, product information and dilutions used for western blot (WB) and immunofluorescence (IF) experiments. **Table S2**. List of genes and primer sequences. **Figure S1**. Caco-2 cells were pre-treated with CFTR inhibitors-172 (10 µM) or GlyH-101 (10 µM) for 30 minutes. Cells were then co-incubated with FAC and erastin for 8 hours and cell viability was assessed by MTS assay (A). C38 cells were pre-treated with CFTR inhibitors for 30 minutes, then incubated in the presence of FAC and erastin for up to 12 hours. Cell death was assessed by incubating cells with SYTOXTM Green nucleic acid stain (125 nM). Cells were imaged every 4 h intervals for 12 hours using the IncuCyte^®^ ZOOM Live-Cell Analysis System. Cell death was measured by counting maximum SYTOX^TM^ Green positive cells normalised to starting cell confluence (B) from images generated by IncuCyte ZOOM software; representative images are shown. Scale bar indicates 300 µm (C). **Figure S2**. Full representative western blot images for TFR1, GPX4, NCOA4, ferritin and β-actin. **Figure S3**. Representative (n=3) immunoblot images and quantification of β-actin normalised ferritin, NCOA4 and TFR1 from AECs treated with DMSO or FAC and erastin for 8 hours. *p<0.05 and **p<0.01 for statistical analysis of the indicated groups. **Figure S4**. MLKL membrane localisation assessed by immunofluorescence in IB3-1 cells treated with FAC (100 µM) and erastin (10 µM). **Figure S5**. C38 (WT) and IB3-1 (CF) cells were treated with FAC (100 µM) and erastin (Eras) (10 µM) in the presence or absence of DFO (100 µM), Fer-1 (2 µM), Nec-1s (50 µM) or NSA (10 µM) for 8 hours and lipid peroxidation were assayed. The lipophilic redox-sensitive dye C11-BODIPY 581/591 shifts its fluorescence from red to green in response to oxidation. Representative images of C38 (A) and IB3-1 (B) cells and quantification are shown (C).

## Data Availability

The datasets used and/or analysed during the study are available from the corresponding author on reasonable request.
