# Supplementary material for: Increased susceptibility of cystic fibrosis airway epithelial cells to ferroptosis
Source: Biol Res. 2021 Dec 13;54:38. doi: 10.1186/s40659-021-00361-3 (PMC8670191; doi:10.1186/s40659-021-00361-3)
Supplement: Supplementary file 1 — Additional file 1: Table S1. List of antibodies, product information and dilutions used for western blot (WB) and immunofluorescence (IF) experiments. Table S2. List of genes and primer sequences. Figure S1. Caco-2 cells were pre-treated with CFTR inhibitors-172 (10 µM) or GlyH-101 (10 µM) for 30 minutes. Cells were then co-incubated with FAC and erastin for 8 hours and cell viability was assessed by MTS assay (A). C38 cells were pre-treated with CFTR inhibitors for 30 minutes, then incubated in the presence of FAC and erastin for up to 12 hours. Cell death was assessed by incubating cells with SYTOXTM Green nucleic acid stain (125 nM). Cells were imaged every 4 h intervals for 12 hours using the IncuCyte® ZOOM Live-Cell Analysis System. Cell death was measured by counting maximum SYTOXTM Green positive cells normalised to starting cell confluence (B) from images generated by IncuCyte ZOOM software; representative images are shown. Scale bar indicates 300 µm (C). Figure S2. Full representative western blot images for TFR1, GPX4, NCOA4, ferritin and β-actin. Figure S3. Representative (n=3) immunoblot images and quantification of β-actin normalised ferritin, NCOA4 and TFR1 from AECs treated with DMSO or FAC and erastin for 8 hours. *p<0.05 and **p<0.01 for statistical analysis of the indicated groups. Figure S4. MLKL membrane localisation assessed by immunofluorescence in IB3-1 cells treated with FAC (100 µM) and erastin (10 µM). Figure S5. C38 (WT) and IB3-1 (CF) cells were treated with FAC (100 µM) and erastin (Eras) (10 µM) in the presence or absence of DFO (100 µM), Fer-1 (2 µM), Nec-1s (50 µM) or NSA (10 µM) for 8 hours and lipid peroxidation were assayed. The lipophilic redox-sensitive dye C11-BODIPY 581/591 shifts its fluorescence from red to green in response to oxidation. Representative images of C38 (A) and IB3-1 (B) cells and quantification are shown (C). [file 40659_2021_361_MOESM1_ESM.docx]

**Supplementary Information**

# Increased susceptibility of cystic fibrosis airway epithelial cells to ferroptosis

Maniam et al.

This file includes:

Supplementary Table 1-2

Supplementary Figure 1-5

**Supplementary Table 1**

Table S1. List of antibodies, product information and dilutions used for western blot (WB) and immunofluorescence (IF) experiments.

| **Antibodies** | **Product number** | **Dilutions** |
| --- | --- | --- |
| GPX4 | 125066 (Abcam) | 1:1000 (WB) |
| NCOA4 | 86707 (Abcam) | 1:1000 (WB) |
| Ferritin-H/L | 75973 (Abcam) | 1:1000 (WB) |
| TFR1 | 214039 (Abcam) | 1:1000 (WB) |
| β-actin | 6276 (Abcam) | 1:10 000 (WB) |
| MLKL | 74921 (Cell Signaling Technology) | 1: 200 (IF) |
| Alexa Fluor Goat anti-rabbit 546 | A11010 (Invitrogen) | 1: 300 (IF) |
| Goat anti-mouse IgG (H+L) secondary antibody | 31430 (Invitrogen) | 1: 10 000 (WB) |
| Goat anti-rabbit IgG (H+L) secondary antibody | 656120 (Invitrogen) | 1: 10 000 (WB) |

**Supplementary Table 2**

Table S2. List of genes and primer sequences.

| **Genes (Human)** | **Forward primers** | **Reverse primers** |
| --- | --- | --- |
| *HPRT* | TGTTGTAGGATATGCCCTTGACT | GGCGATGTCAATAGGACTCCA |
| *PTGS2* | ATGCTGACTATGGCTACAAAAGC | TCGGGCAATCATCAGGCAC |
| *SLC7A11* | TCTCCAAAGGAGGTTACCTGC | AGACTCCCCTCAGTAAAGTGAC |

**Supplementary Figure 1**

A


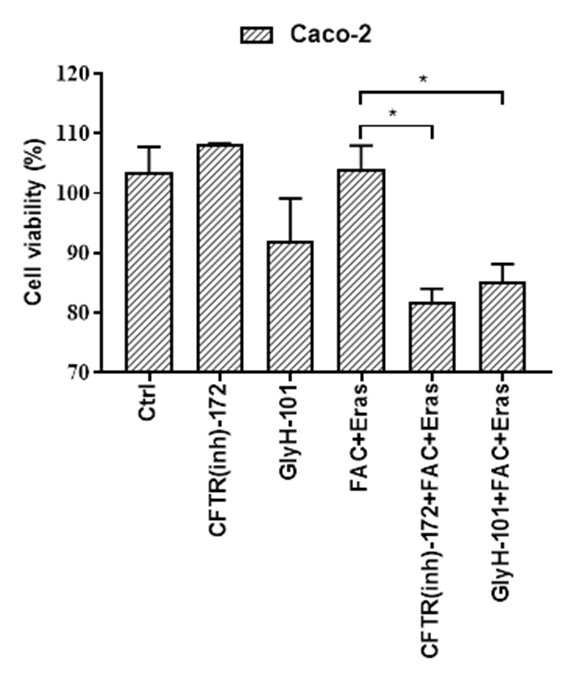


**
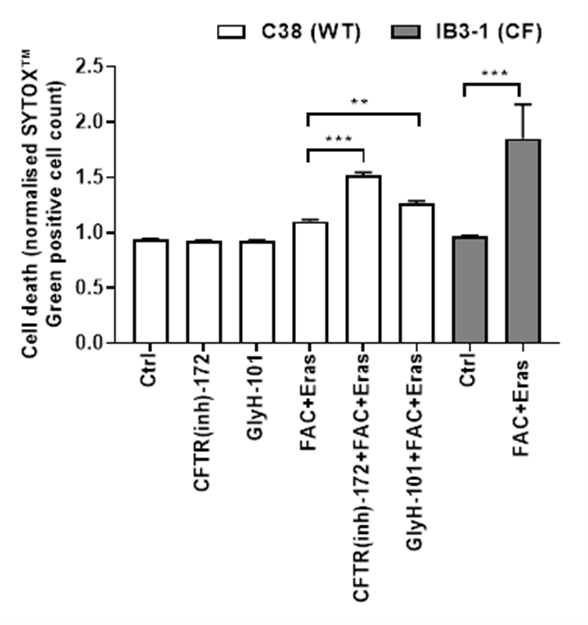
**

B

C

Figure S1. Caco-2 cells were pre-treated with CFTR inhibitors-172 (10 µM) or GlyH-101 (10 µM) for 30 minutes. Cells were then co-incubated with FAC and erastin for 8 hours and cell viability was assessed by MTS assay (A). C38 cells were pre-treated with CFTR inhibitors for 30 minutes, then incubated in the presence of FAC and erastin for up to 12 hours. Cell death was assessed by incubating cells with SYTOX^TM^ Green nucleic acid stain (125 nM). Cells were imaged every 4 h intervals for 12 hours using the IncuCyte^®^ ZOOM Live-Cell Analysis System. Cell death was measured by counting maximum SYTOX^TM^ Green positive cells normalised to starting cell confluence (B) from images generated by IncuCyte ZOOM software; representative images are shown. Scale bar indicates 300 µm (C).

**Supplementary Figure 2**

Figure S2. Full representative western blot images for TFR1, GPX4, NCOA4, ferritin and β-actin. Samples 1: C38 (WT) Control; 2: IB3-1 (CF) control; 3: C38 FAC+Eras and 4: IB3-1 FAC+Eras.

**Supplementary Figure 3**

**
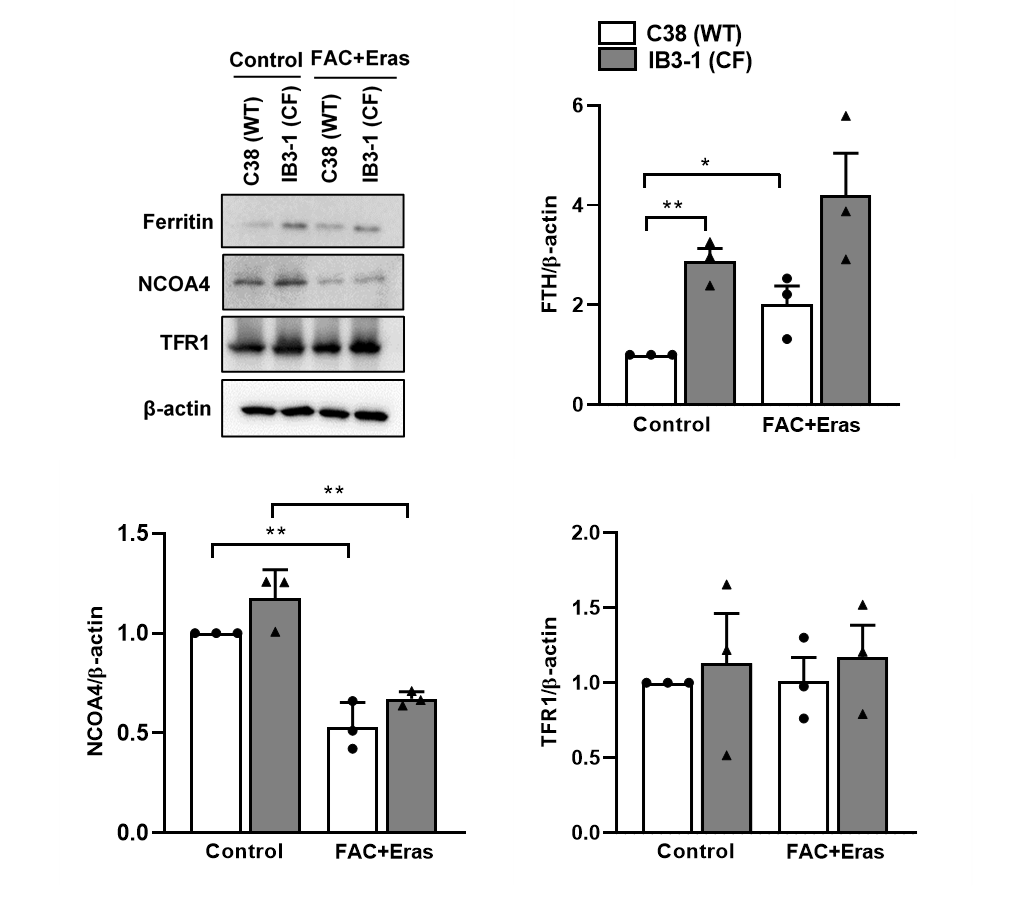
**

Figure S3. Representative (n=3) immunoblot images and quantification of β-actin normalised ferritin, NCOA4 and TFR1 from AECs treated with DMSO or FAC and erastin for 8 hours. *p<0.05 and **p<0.01 for statistical analysis of the indicated groups.

.

**Supplementary Figure 4**


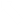


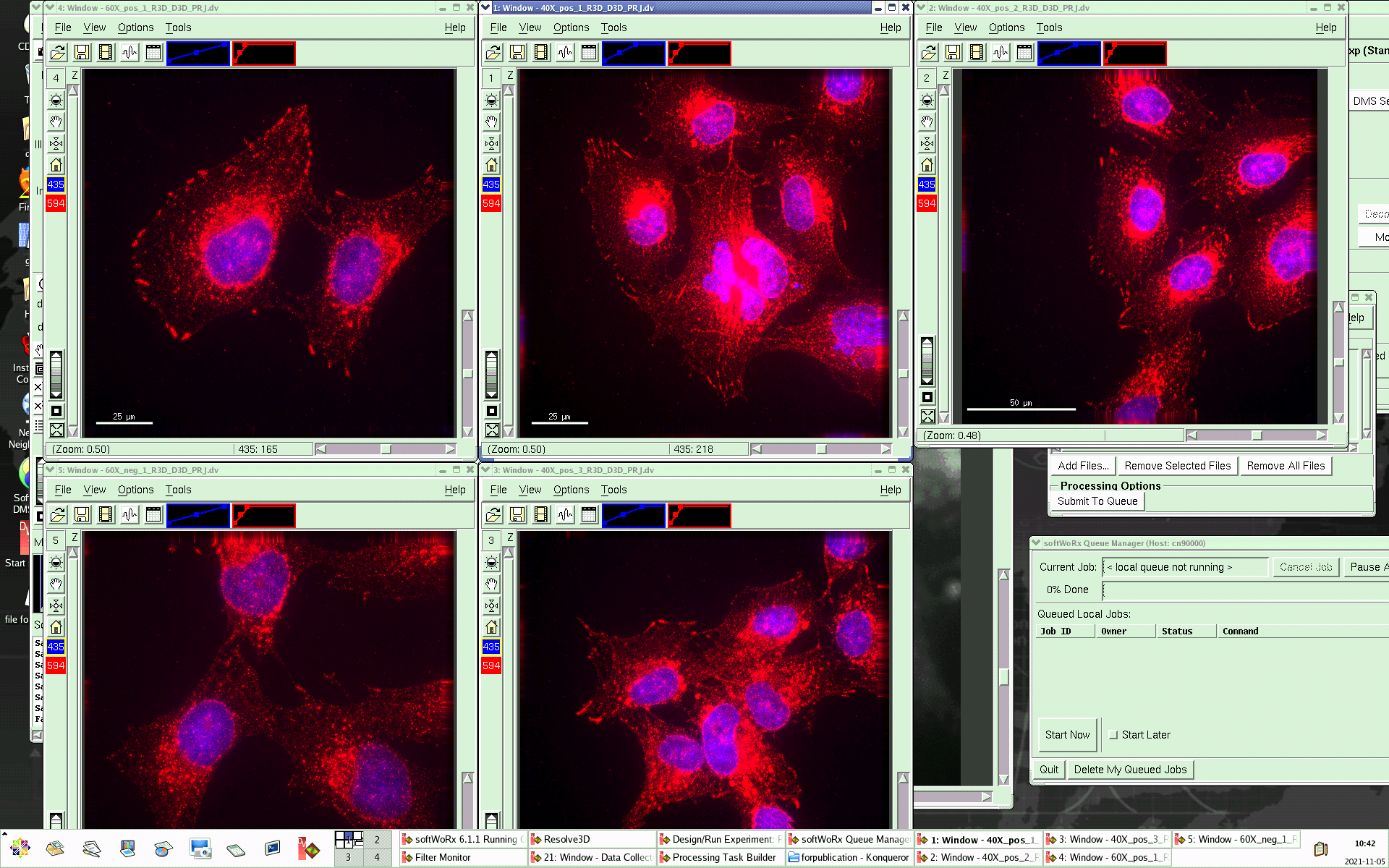


**50 µm**

**m**

Figure S4. MLKL membrane localisation assessed by immunofluorescence in IB3-1 cells treated with FAC (100 µM) and erastin (10 µM).

**Supplementary Figure 5**

**
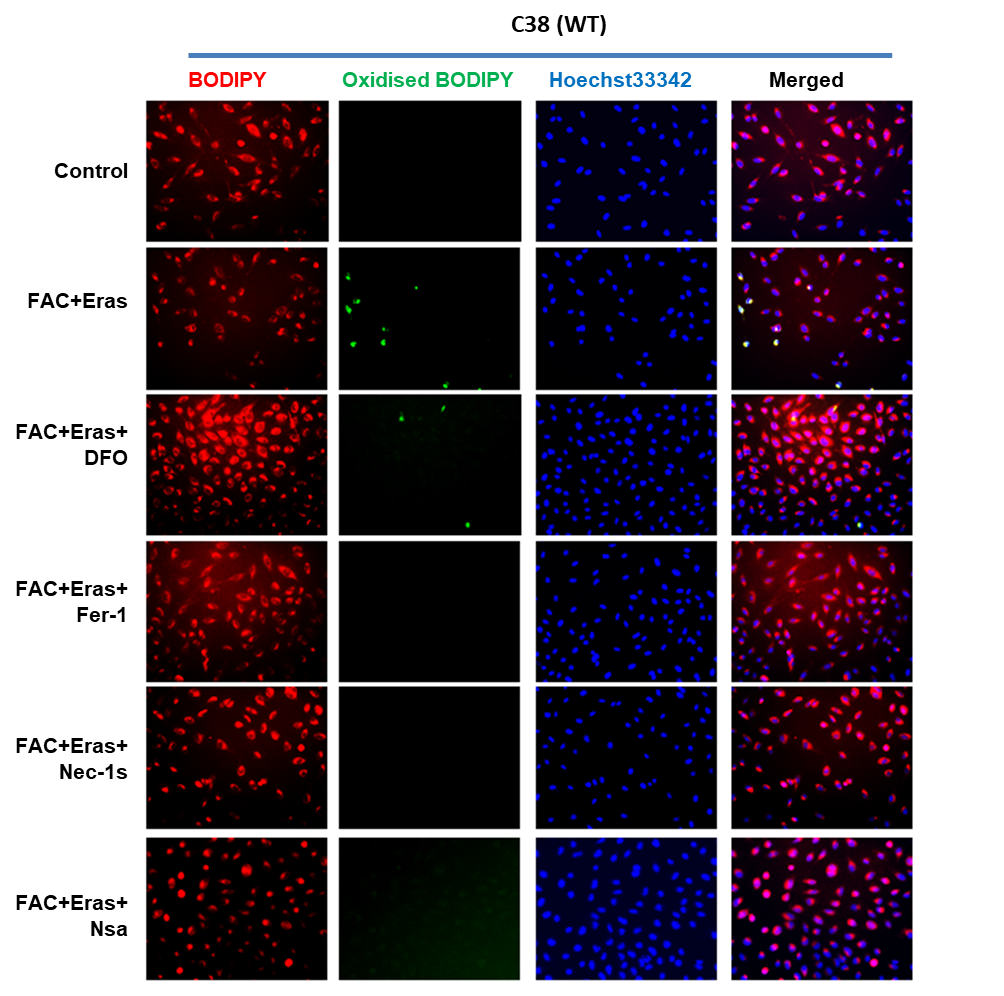
**

A


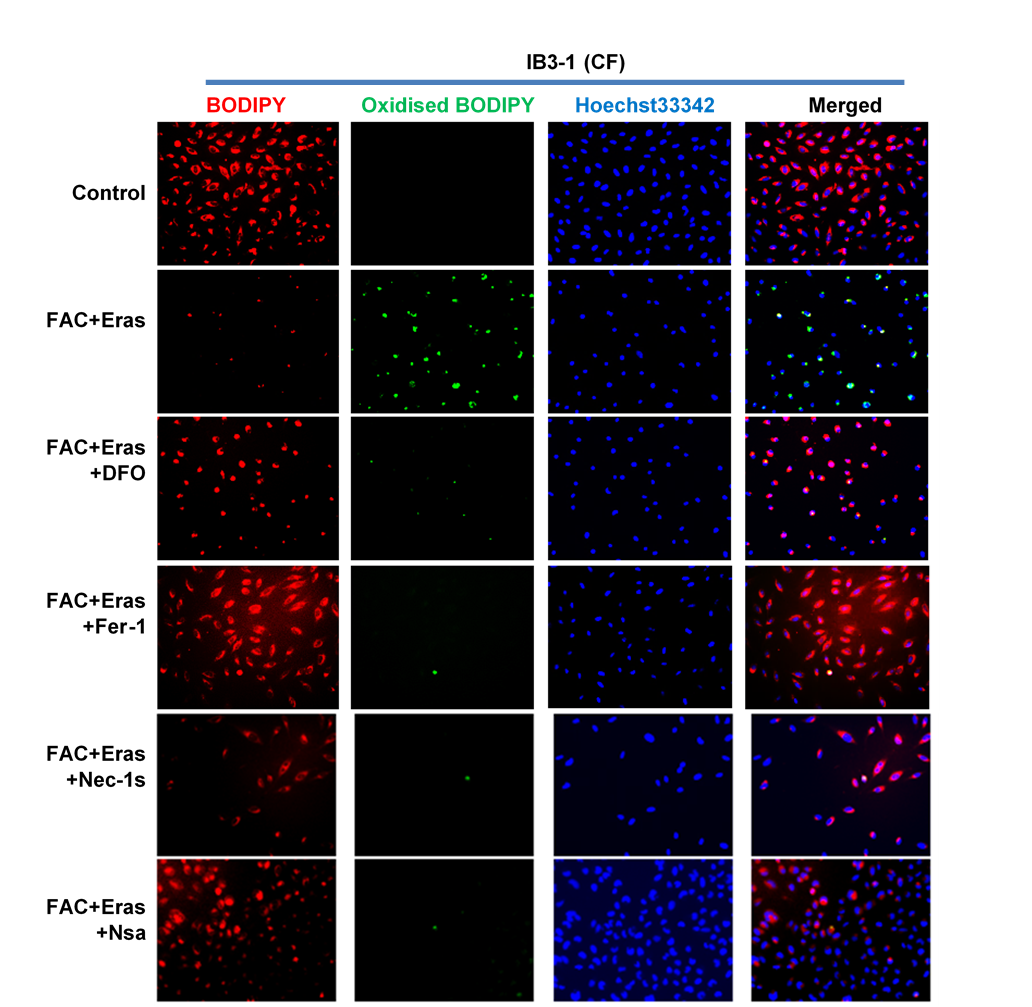


B


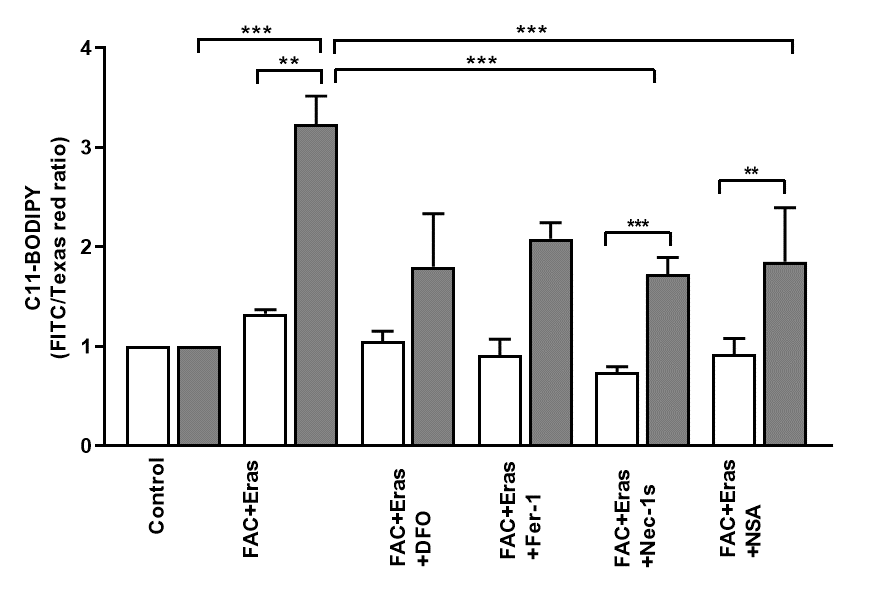


C

Figure S5. C38 (WT) and IB3-1 (CF) cells were treated with FAC (100 µM) and erastin (Eras) (10 µM) in the presence or absence of DFO (100 µM), Fer-1 (2 µM), Nec-1s (50 µM) or NSA (10 µM) for 8 hours and lipid peroxidation were assayed. The lipophilic redox-sensitive dye C11-BODIPY 581/591 shifts its fluorescence from red to green in response to oxidation. Representative images of C38 (A) and IB3-1 (B) cells and quantification are shown (C).
